# Maladjustment of pressure settings of a programmable shunt valve by electromagnetic door locks in Forensic Psychiatry – a case report

**DOI:** 10.1192/j.eurpsy.2022.1550

**Published:** 2022-09-01

**Authors:** C. Licht, R. Weisser, C. Schloegl

**Affiliations:** 1 Paracelsus Medical University Clinic Nuremberg, Psychiatry And Psychotherapy, Nuernberg, Germany; 2 medbo Regensburg, Forensic Psychiatry, Regensburg, Germany

**Keywords:** maladjustment, Codman-Hakim programmable shunt valve, magnetic field, case report

## Abstract

**Introduction:**

Maladjustments and failures of programmable ventriculo-peritoneal shunts have been reported in patients encountering powerful electromagnetic fields, e. g. MRI.

We describe the case of a 53-year old man treated for hydrocephalus with a programmable Codman-Hakim shunt valve. During his hospitalization in Forensic Psychiatry, the patient’s valve pressure setting changed randomly despite frequent reprogramming and surveillance.

**Objectives:**

Maladjustments and failures of programmable ventriculo-peritoneal shunts have been reported in cases in which patients have encountered powerful electromagnetic fields, e.g., MRI. Through a case, this study shows easy maladjustment of a Codman-Hakim programmable valve also by small magnetic fields from everyday life.

**Methods:**

A 53-year old man presented with periventricular hydrocephalus due to aqueductal stenosis. The patient was treated with a left ventriculo-peritoneal Codman-Hakim programmable shunt valve. During his hospitalization in Forensic Psychiatry, the patient’s valve pressure setting changed randomly, presumably by walking through electromagnetically controlled doors of a hospital ward. With a test dummy, changes in pressure settings were tracked.

**Results:**

Both - pressure settings of the patient’s Codman-Hakim programmable valve as well as pressure settings of a new valve - were unwantedly modified simply by walking through standard doors in a hospital ward.

**Conclusions:**

Thus already weak magnetic fields (< 200 mT) might cause changes in the pressure settings of programmable shunt valves and therefore lead to maladjustment. Patients should be informed and pay attention to using everyday life’s devices, like rod magnets or mobile phones.

**Disclosure:**

No significant relationships.

